# Peroxiredoxin-1 promotes cell proliferation and metastasis through enhancing Akt/mTOR in human osteosarcoma cells

**DOI:** 10.18632/oncotarget.23662

**Published:** 2017-12-23

**Authors:** An-Lie Cai, Wei Zeng, Wei-Liang Cai, Jing-Ling Liu, Xue-Wen Zheng, Ying Liu, Xiang-Cheng Yang, Yi Long, Jie Li

**Affiliations:** ^1^ Department of Orthopedics Surgery, Central Hospital of Zhuzhou city and The Affiliated Zhuzhou Hospital of Xiangya Medical College of Central South University, Zhuzhou, China; ^2^ Department of Orthopedics Surgery, Second Xiangya Hospital Central South University, Changsha, Hunan, China; ^3^ Department of Nephrology, Central Hospital of Zhuzhou City and Affiliated Zhuzhou Hospital of Xiangya Medical College of Central South University, Zhuzhou, China

**Keywords:** peroxiredoxin-1, osteosarcoma, metastasis, cell migration

## Abstract

Osteosarcoma is characterized by high propensity for metastasis, especially to the lung, which is the main cause of death. Peroxiredoxin-1 (PRDX1) plays significant roles in multiple processes of initiation and progression of tumorogenesis. However, whether PRDX1 participates in metastasis of osteosarcoma remains unknown. Here, we demonstrate that PRDX1 overexpressed in osteosarcoma tissues comparing to adjacent non-tumor tissues. Two independent cohorts of patients showed high level of PRDX1 correlated with clinicopathological features such as larger tumor size and advanced tumor metastasis stage. While patients with high PRDX1 level have poor prognosis. Notably, expression level of PRDX1 especially increased in lung lesion of osteosarcoma patients, indicating that PRDX1 may promote lung metastasis. Ectopic expression of PRDX1 promotes osteosarcoma cell migration and metastasis *in vitro* and *in vivo*, whereas knockdown of PRDX1 expression suppresses cell metastatic behaviors such as invasion and migration. Furthermore, we found that PRDX1 promotes cells metastasis through enhancing Akt/mTOR signal pathway. Taken together, our findings prove the important role of PRDX1 in the molecular etiology of osteosarcoma and suggest that PRDX1 may be a novel prognostic biomarker and therapeutic target for osteosarcoma.

## INTRODUCTION

Osteosarcoma, one of the most common primary bone tumors in children and adolescent, most often localized in the metaphysis of adolescent long bones [1]. The survival of patients is largely dependent on the malignant stage and whether there is lung metastasis. Patients without lung metastasis have five-year survival rate range from 50% to 70% after radical tumor resection, whereas those with lung metastasis have less than 20% five-year survival rate [2]. Despite remarkably improved diagnostic and treatment strategies, patients still have high incidences of tumor recurrence and metastasis, especially lung metastasis [3]. Previous studies identified a number of tumor suppressor or oncogenes, such as p53 and Rb, which play significant roles in tumorogenesis. But the molecular mechanism and cellular process of metastasis is still poorly understood [4].

Peroxiredoxins (PRDXs) have received considerable attention in recent years as a new family of thiol-specific antioxidant proteins. PRDXs exert their protective role in cells through anti-oxidation via their peroxidase activity. PRDXs are a ubiquitous family of redox-regulating proteins, which are essential for cell metabolism and cell viability and act as a regulator of redox signaling [5]. Redox signaling is a critical signaling pathway involved in the regulation of cell metabolism, cell growth, immune response and variety of other physiological functions. Studies demonstrated that PRDXs involved in several type of cancers, neurodegenerative diseases and inflammatory related diseases [6]. The hyper-proliferative property of cancer cells is known to be associated with increased production of intracellular reactive oxygen species (ROS) and couple of studies have shown that family member of PRDXs inhibits or promote the development and progression of cancer [7, 8].

PRDX1, which belongs to the PRDXs family, is composed of thiol-specific antioxidant enzyme that reduces peroxynitrite and is associated with mitigation of oxidative damage [9]. The function of PRDX1 in carcinogenesis is controversial. Some groups found that PRDX1 may promote tumor development but other groups identified the tumor suppression function in breast cancer and esophageal squamous cell carcinoma [10]. Substantial data indicate that PRDX1 is also implicated in metastatic behavior, cell spreading and migration [11]. Elevated PRDX1 expression in lung cancer is associated with cell growth and metastasis [12]. However, the function of PRDX1 in progression and metastasis of osteosarcoma is unknown.

In present study, we found overexpression of PRDX1 was frequently observed in osteosarcoma tissues and human cell lines. Expression level of PRDX1 is highly correlated with prognosis of osteosarcoma patients. Knockdown of PRDX1 suppress cell growth and invasion *in vitro* and *in vivo*. Notably, knockdown of PRDX1 inhibit lung metastasis of osteosarcoma cells *in vivo*. Our results suggest that aberrant expression of PRDX1 is critical for the metastasis of human osteosarcoma.

## RESULTS

### PRDX-1expression is frequently up-regulated in human osteosarcoma tissues and cell lines

**Figure 1:**
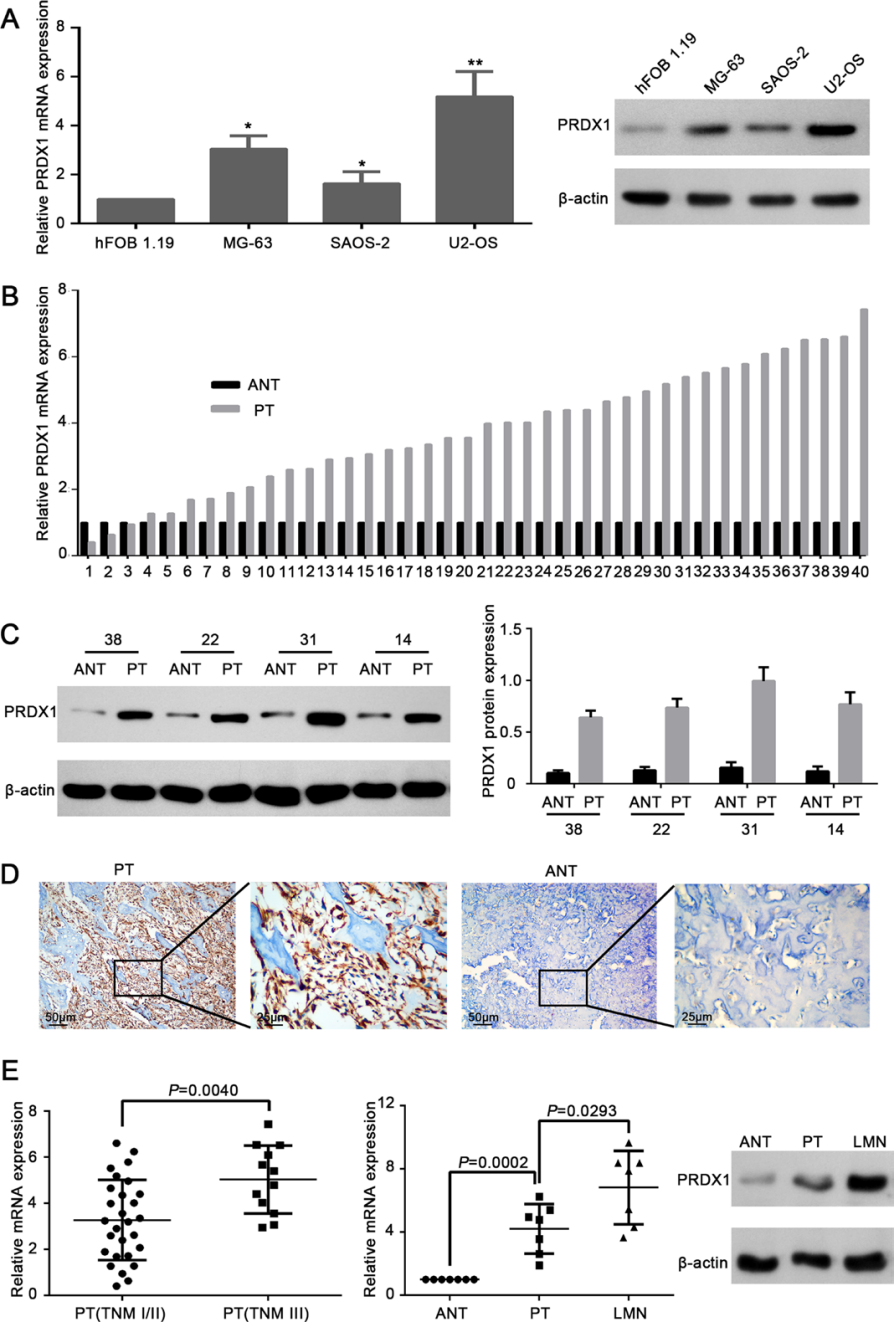
Expression level of PRDX1 elevates in human osteosarcoma tissues and cell lines (**A**) PRDX1 was overexpressed in osteosarcoma cell lines. Expression level of PRDX1 increased significantly in osteosarcoma cell lines MG-63, SAOS-2 and U2-OS when comparing to hFOB 1.19(normal osteoblast cells. Expression level was examined by qRT-PCR (left panel) and western blot (right panel). (**B**) qRT-PCR showed the mRNA level of PRDX1 was higher in PTs than in corresponding ANT. PT, primary tumor tissue; ANT, adjacent non-tumor tissue (*n* = 30). (**C**) Representative images of Western blot. Results showed the protein expression level of PRDX1 in PTs was higher than in corresponding NCBTs (*n* = 30). (**D**) Representative images of immunohistochemistry (IHC) staining of PRDX1 in osteosarcoma tissues. PTs exhibited strong positive signal than corresponding ANT. Magnification of images, × 400. (**E**) PRDX1 mRNA level was higher in TNM stage III (*n* = 12) than in TNM stage I/II (*n* = 30). (**F**) PRDX1 level was increased both at mRNA (left panel) and Protein level (right panel) in LMNs than corresponding NCBTs from the same patient (*n* = 7). LMNs, lung metastatic nodules.

Comparing to the normal cell line hFOB 1.19, PRDX1 mRNA and protein level was elevated in osteosarcoma cell lines MG-63, U2-OS, and SAOS-2 (Figure 1A). To get the correlation between PRDX1 and osteosarcoma, we screened the PRDX1 expression pattern in 40 pairs of fresh osteosarcoma tissue specimens. Importantly, the levels of PRDX1 mRNA (Figure 1B) and protein (Figure 1C) were dramatically increased in primary tumor tissues (PTs) than in corresponding adjacent non-tumor tissues (ANTs). Furthermore, immunohistochemical (IHC) staining also showed the expression of PRDX1 protein was significantly higher in PT than in ANTs (Figure 1D). Surprisingly, PRDX1 level was higher in primary tumors of TNM stage III than that of TNM stage I/II (Figure 1E). Notably, PRDX1 expression was higher in lung metastatic nodules (LMN) than in their corresponding PTs and ANTs from same patients (*n* = 7). Interestingly, the expression pattern gradually increased from NCBTs, PTs to LMN (Figure 1F), indicating PRDX1 may contribute to lung metastasis.

**Figure 2:**
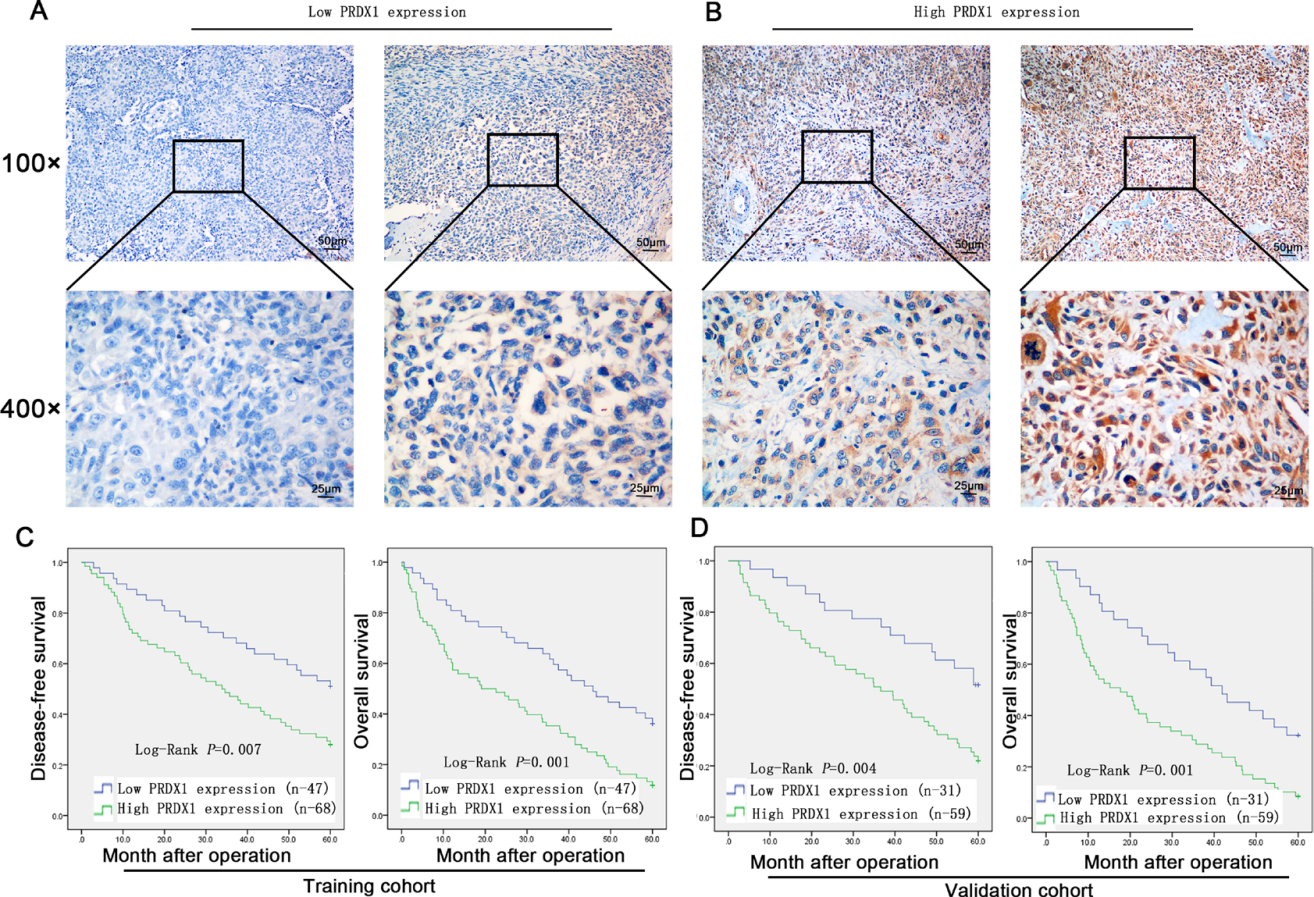
Expression level of PRDX1 is associated with poor prognosis of osteosarcoma patients (**A** and **B**) Representative images of PRDX1 IHC staining in osteosarcoma tissues. Expression level was scored base on IHC staining. Osteosarcoma patients dichotomized into low PRDX1 expression group (Score < 3; A) and high PRDX1 expression group (Score≥ 3; B). (**C** and **D**) Kaplan-Meier survival curves show that disease-free survival (DFS; left column) and overall survival (OS; right column) in training cohort. Log-rank test showed that osteosarcoma patients with high level of PRDX1 had significantly shorter DFS and OS than those with low level of PRDX1.

To evaluate the prognostic value of PRDX1, IHC was used to measure the PRDX1 level in two cohorts of patients including training cohort (*n* = 110, Table 1) and validation cohort (*n* = 90, Table 1). In training cohort, patients were divided into PRDX1 low (Figure 2A) and the PRDX1 high (Figure 2B). We found that 60.0% patients exhibited high level of PRDX1 and the level of PRDX1 were significantly associated with tumor size (*P* < 0.001), high malignant grade (*P* < 0.01), and advanced TNM stage (*P* < 0.01), but it was not associated with patient gender (*P* = 0.584), age (*P* = 0.856), anatomical localization of tumor (*P* = 0.291) and ALP level (*P* = 0.880) (Table 1). Most importantly, log-rank test showed that osteosarcoma patients with high level of PRDX1 had significantly shorter disease free survival (DFS) and overall survival (OS) than those with low level of PRDX1 (Figure 2C). Remarkably, expression level of PRDX1 was found to be the independent predictors for DFS (*P* = 0.001 in Univariate-analysis and *P* = 0.006 in multivariate-analysis; Table 2) and OS (*P* = 0.008 in Univariate-analysis and *P* = 0.007 in multivariate-analysis; Table 2) in osteosarcoma. Furthermore, this was confirmed in validation cohort of 90 osteosarcoma patients. Expression level of PRDX1 was validated to be the predictors for DFS (*P* = 0.002 in Univariate-analysis and *P* = 0.001 in multivariate-analysis; Table IV) and OS (*P* = 0.005 in Univariate-analysis and *P* = 0.004 in multivariate-analysis; Table 3) in osteosarcoma. Taking together, these data indicates that PRDX1 may contribute to osteosarcoma tumorogenesis and metastasis.

**Table 1:** Correlations of PRDX1 expression with clinicopathological variables of osteosarcoma in training and validation cohort

Variables	Training Cohort				Validation Cohort			
	PRDX1 Expression				PRDX1 Expression			
	*N*	Low	High	*P* value	No.	Low	High	*P* value
**Gender**								
Male	75	31	44	0.676	67	22	45	0.584
Female	35	13	22		23	9	14	
**Age (years)**								
< 45	57	20	37	0.275	54	19	35	0.856
> 45	53	24	29		36	12	24	
**Anatomical site**								
Femur/Tibia	89	36	53	0.843	67	21	46	0.291
Elsewhere	21	8	13		23	10	13	
**Tumor size (cm)**								
< 8	50	30	20	**< 0.001**	41	22	19	**< 0.001**
> 8	60	14	46		49	9	40	
**ALP level(U/L)**								
< 150	44	16	28	0.525	31	11	20	0.880
> 150	66	28	38		59	20	39	
**Pathological fracture**								
Absent	91	41	50	**0.018**	69	27	17	**< 0.001**
Present	19	3	16		21	4	42	
**Ennecking grade**								
2a	61	30	31	**0.028**	47	23	24	**0.002**
2b	49	14	35		43	8	35	
**Histologic grade**								
Low	65	33	32	**0.006**	47	24	23	**0.001**
High	45	11	34		44	8	36	
**TNM stage**								
I/II	62	32	30	**0.005**	47	25	22	**< 0.001**
III	48	12	36		43	6	37	

**Table 2:** Univariate and multivariate analysis of disease-free survival (dfs) and overall survival (os) in training cohort

Variables		DFS				OS			
		Univariate analysis		Multivariate analysis		Univariate analysis		Multivariate analysis	
		HR (95% CI)	*P*	HR (95% CI)	*P*	HR (95% CI)	*P*	HR (95% CI)	*P*
**Tumor size (cm)**	< 8 vs. > 8	2.041 (1.340–3.108)	**0.001**	1.831 (1.173–2.860)	**0.008**	2.115 (1.329–3.367)	**0.002**	1.723(1.045–2.841)	**0.033**
**Histologic grade**	Low vs. High	1.606 (1.051–2.455)	**0.029**	1.620(0.985–2.666)	0.058	1.669 (1.049–2.657)	**0.031**	1.788 (1.109–2.881)	**0.017**
**TNM stage**	I/II vs. III	2.446(1.599-3.740)	**< 0.001**	1.976(1.280-3.052)	**0.002**	2.139(1.341–3.412)	**0.001**	1.951(1.216-3.129)	**0.006**
**PRDX1 expression**	Low vs. High	2.114 (1.359–3.289)	**0.001**	1.845 (1.191–2.856)	**0.006**	1.951 (1.187–3.208)	**0.008**	1.938(1.197–3.138)	**0.007**

**Table 3:** Univariate and multivariate analysis of disease-free survival (dfs) and overall survival (os) in validation cohort

Variables		DFS				OS			
		Univariate analysis		Multivariate analysis		Univariate analysis		Multivariate analysis	
		HR (95% CI)	*P*	HR (95% CI)	*P*	HR (95% CI)	*P*	HR (95% CI)	*P*
Tumor size (cm)	< 8 vs. > 8	2.101 (1.315–3.356)	**0.002**	1.860(1.114–3.106)	**0.018**	2.239 (1.344–3.731)	**0.002**	1.890 (1.102–3.241)	**0.021**
Histologic grade	Low vs. High	1.433 (0.909–2.258)	0.121		**NA**	2.375 (1.362–4.142)	**0.002**	1.596 (0.876-2.906)	0.126
TNM stage	I/II vs. III	2.197 (01.380-3498)	**0.001**	2.130 (1.222-3.711)	**0.008**	2.158 (1.300-3.582)	**0.003**	1.943 (1.145-3.298)	**0.014**
PRDX1 expression	Low vs. High	2.246 (1.350–3.735)	**0.002**	2.213 (1.374–3.564)	**0.001**	2.330 (1.297–4.184)	**0.005**	2.261 (1.288–3.970)	**0.004**

### PRDX1 promotes invasion and proliferation of osteosarcoma cells *in vitro*

**Figure 3:**
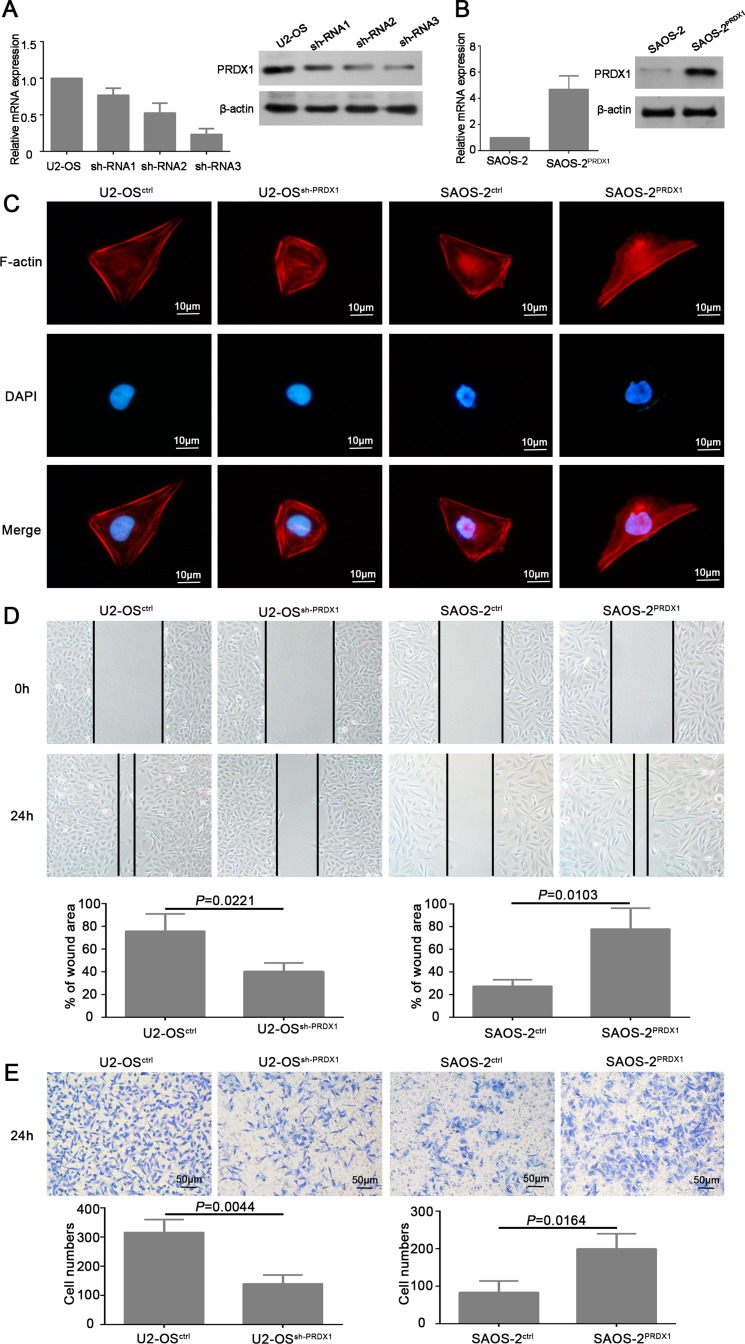
PRDX1 promotes migration and invasion of osteosarcoma cells *in vitro* (A and B) Establishment of PRDX1 knockdown and overexpression stable cells line. U2-OS was transfected with shRNA plasmids (**A**) and PRDX1 overexrpession plasmids (**B**). PRDX1 expression level was examined by qRT-PCR and western blot after successfully generating stable cell line. (**C**) Manipulation of PRDX1 changes cell morphology of osteosarcoma cells. F-actin filaments were visualized by using rhodamine-phalloidin. (**D**) Wound-healing assays showed that knockdown of PRDX1 suppress cell motility and invasion when comparing with control cells (left panel). Meanwhile, overexpression of PRDX1 promotes wound healing capacity when comparing with control cells. (**E**) Transwell assays showed that knockdown of PRDX1 suppresses cell migration when comparing with control cells (left panel). Meanwhile, overexpression of PRDX1 promotes cell migration in comparing to control cells.

To determine the roles of PRDX1 in osteosarcoma invasion and metastasis, we established stable cell line of PRDX1 knockdown (U2-OS^shPRDX1^), PRDX1 overexpression (SAOS-2^PRDX1^), and scramble control (Figure 3A and 3B). Several studies proven that cell morphology changing and cytoskeleton re-organization are the important process for cell migration and invasion [13, 14]. The cell cytoskeleton was visualized by staining of F-actin. Our results showed that knockdown of PRDX1 cause stress fiber-like structures disappearance and regression of cell morphology (Figure 3C, left two panel). Overexpression of PRDX1 induced obvious reorganization of actin cytoskeleton (Figure 3C, right two panel).

**Figure 4:**
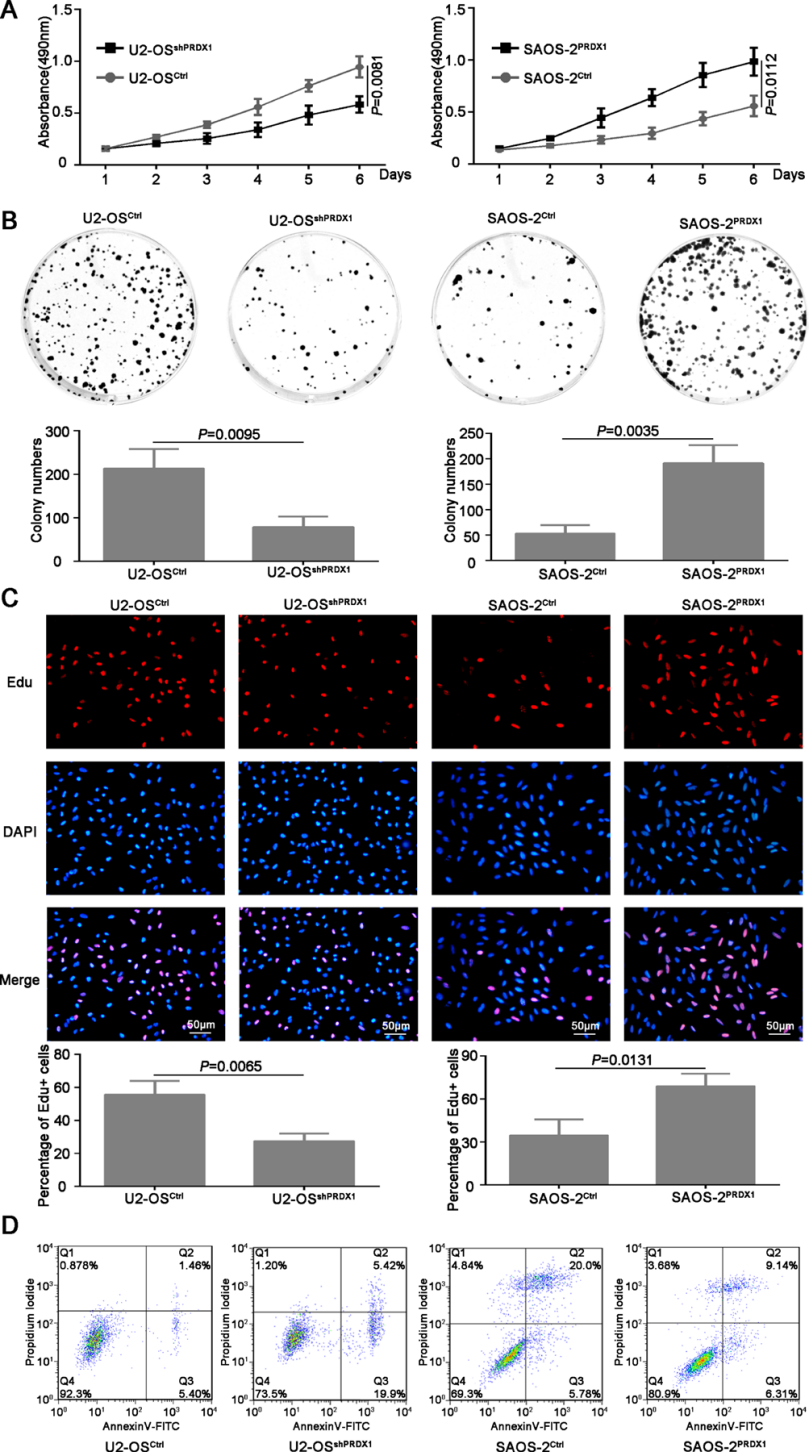
PRDX1 promotes proliferation of osteosarcoma cells *in vitro* (**A**) MTT was used to measure the proliferation ability of U2-OS^shPRDX1^ and U2-OS^Control^, SAOS-2^PRDX1^ and SAOS-2^Vector^ cells. (**B**) The colony formation assay shown that knockdown of PRDX1 inhibits colony formation (left panel) and overexpression of PRDX1 promotes colony formation (right panel). (**C**) Cells were labeled with EdU for 3 hrs and the results revealed that the EdU positive cells were higher in osteosarcoma cells with relative higher PRDX1 level. (**D**) Cell apoptosis was measured by PI/Annexin V. Knockdown of PRDX1 induce cell apoptosis (left panel) but overexpression of PRDX1 suppress cell apoptosis (right panel).

Furthermore, we determined if PRDX1 could promote the invasion of osteosarcoma cells. As shown in Figure 3D, knockdown of PRDX1 dramatically inhibits cell migration, whereas overexpression of PRDX1 promotes cell migration. Transwell assays showed that, compared to their corresponding control cells, an obvious decrease of invaded cells was observed in U2-OS^shPRDX1^ group, but a significant increase of invaded cells was observed in SAOS-2^PRDX1^ group (Figure 3E). In addition, we observed that knockdown of PRDX1 dramatically suppress cells proliferation, while overexpression of PRDX1 significantly accelerated cells proliferation (Figure 4A–4B). EdU Click-assay revealed that the percentage of EdU positive cells were increased in osteosarcoma cells with ectopic PRDX1 expression (Figure 4C). Moreover, knockdown of PRDX1 induce cell apoptosis and overexpression of PRDX1 protect cells against cell death (Figure 4D). These data support a metastasis-promoting role of PRDX1 in osteosarcoma.

### PRDX1 promotes tumor growth and metastasis of osteosarcoma cells *in vivo*

**Figure 5:**
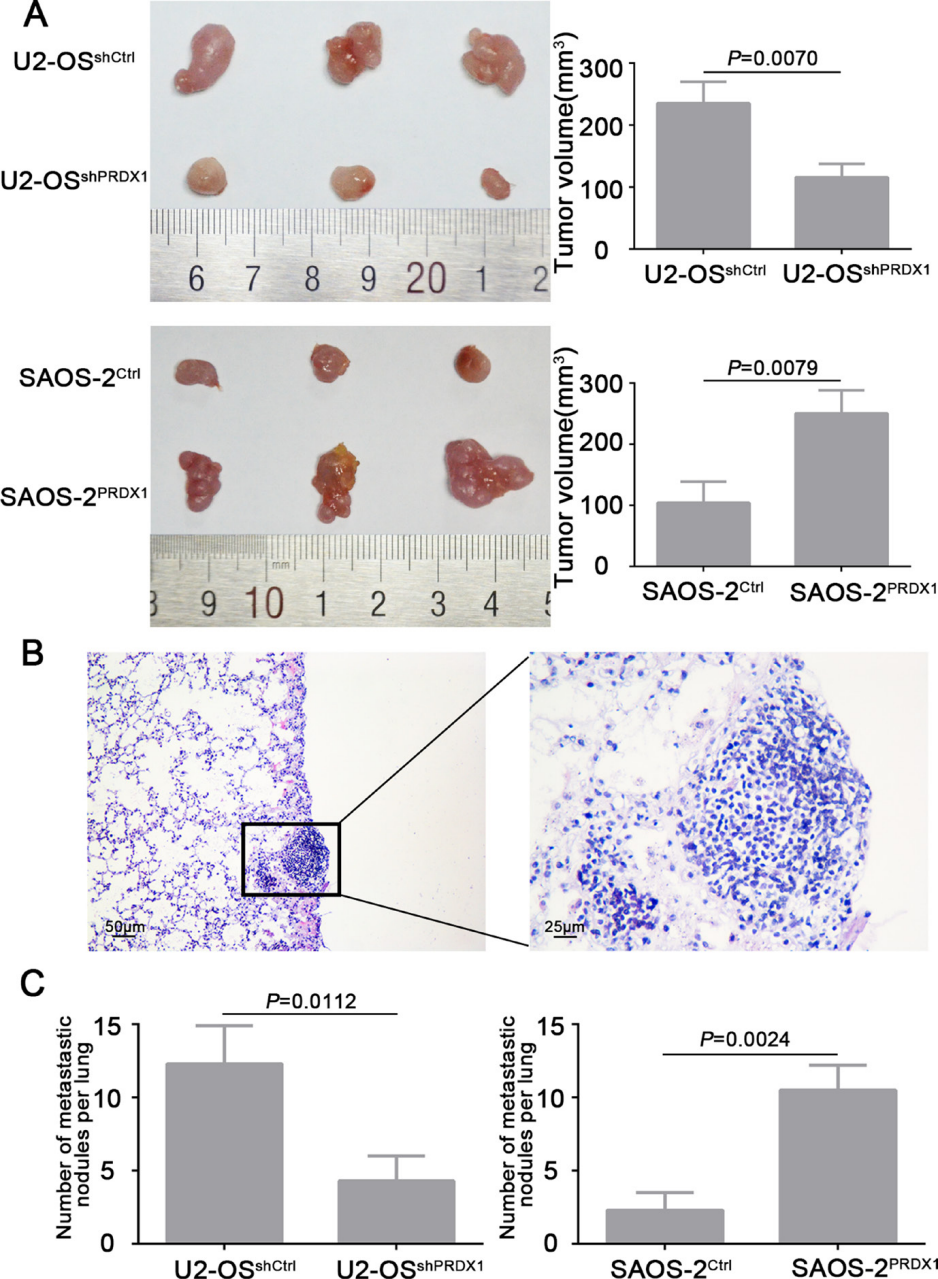
PRDX1 promotes osteosarcoma cells growth and metastasis *in vivo* (**A**) Subcutaneous xenograft model was used to measure the cell growth *in vivo*. U2-OS^shPRDX1^ or U2-OS^Control^ cells and SAOS-2^PRDX1^ or SAOS-2^Vector^ cells were injected into the flank of nude mice. Resulted showed that knockdown of PRDX1 suppresses tumor growth (top panel) and overexpression of PRDX1 promotes tumor growth *in vivo* (bottom panel). (**B**) Representative image of metastatic lesion in lung caused by overexpression of PRDX1. (**C**) The number of lung metastatic nodules was quantified. Knockdown of PRDX1 dramatically suppresses tumor cells metastasize to lung (left panel). In turn, overexpression of PRDX1 enhances the ability of tumor cells metastasize to lung (Right panel). Magnification of images, × 100.

To verify the results that PRDX1 promotes cell growth and invasion *in vitro*, we evaluate the role of PRDX1 *in vivo* by using subcutaneous xenograft and metastasis model. We observed that nude mice injected with U2-OS^shPRDX1^ cells formed a dramatically smaller tumor than U2-OS^Control^ cells (Figure 5A, upper panel). And overexpression of PRDX1 promotes cell growth *in vivo* (Figure 5A, bottom panel). To test the hypothesis that PRDX1 might promote the metastatic ability of osteosarcoma cells *in vivo,* we inject the cells with overexpressed or down-regulated PRDX1 into the tail vein of nude mice (Figure 5B). Consistently, nude mice injected with U2-OS^shPRDX1^ cells had smaller size and fewer number of lung metastasis lesion than those injected with U2-OS^Control^ cells (Figure 5C, left panel). However, the number of lung metastasis lesion in nude mice injected with SAOS-2^PRDX1^ cells dramatically increased than those injected with SAOS-2^Vector^ cells (Figure 5C, right panel). In all, we demonstrated that PRDX1 have the potential of enhancing invasion and metastasis of osteosarcoma cells *in vivo.*

### PRDX1 promotes metastasis and proliferation of osteosarcoma through increasing phosphorylation of Akt/mTOR

**Figure 6:**
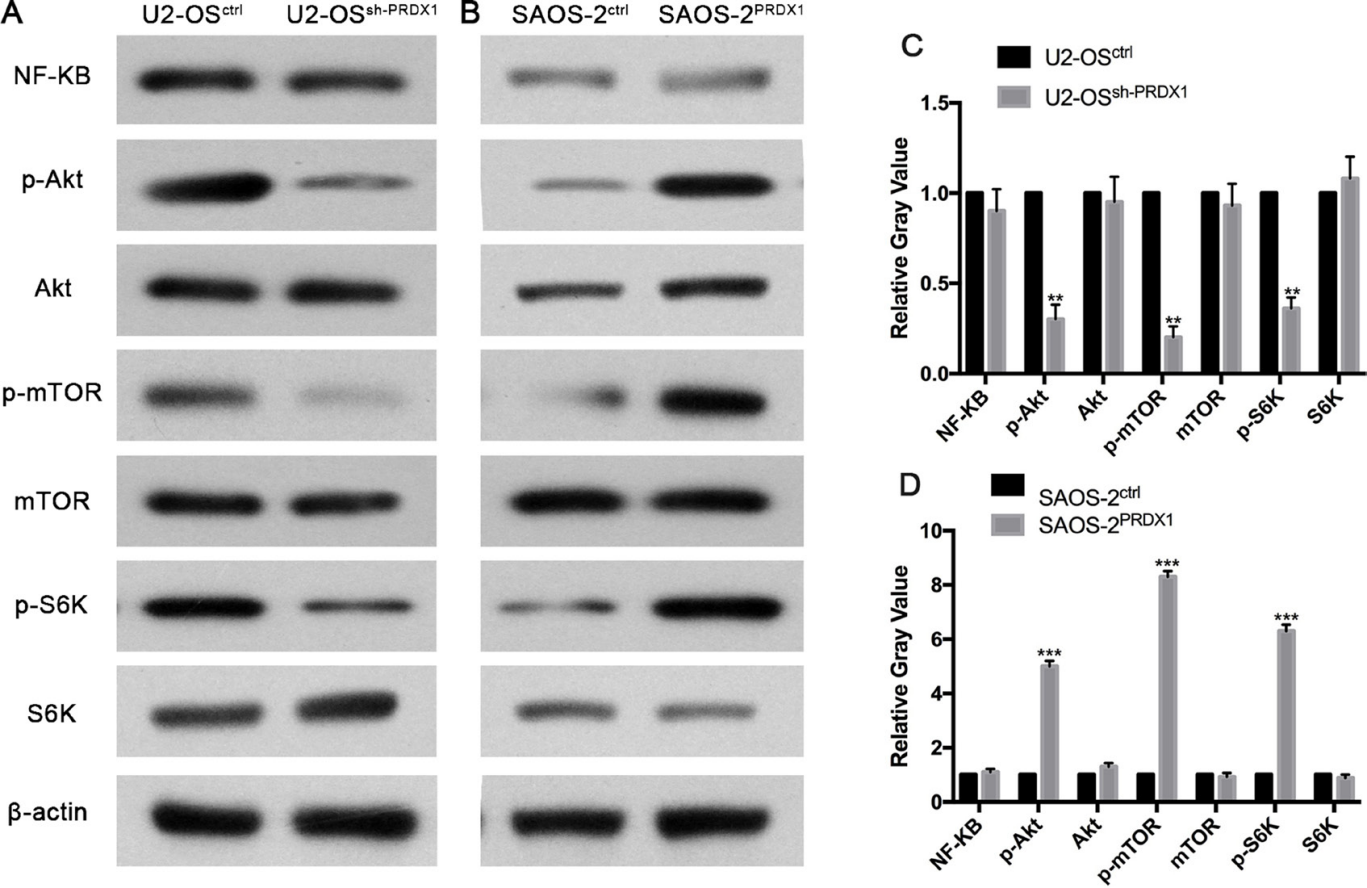
PRDX1 enhances metastasis of osteosarcoma through Akt/mTOR/S6K pathway The levels of key members of Akt/mTOR/S6K signaling were examined by western blot. As the results shown, p-AKT (S473), p-mTOR(S2448) and pS6K decreased after down regulation of PRDX1(left panel) or increase after overexpression of PRDX1(right panel). Optic density was quantified by Bandscan software (BioRad, Hercules, CA) and defined as the ratio of target protein relative to β-actin.

To define the mechanisms by which PRDX1 promotes invasion and metastasis, we tried to identify potential signal pathway regulated by PRDX1 in osteosarcoma. Previous study reported that PRDX1 was functionally involved in Akt/mTOR in esophageal squamous cell carcinoma [15]. And also, Akt/mTOR pathway is critical for tumor distant metastasis in osteosarcoma mouse model and cultured cells [16, 17]. Here, we found that knockdown of PRDX1 suppress phosphorylation of Akt/mTOR/S6K in osteosarcoma cells, and overexpression of PRDX1 increase phosphorylation of Akt/mTOR/S6K. While the total protein level of Akt, mTOR, and S6K did not increased or decreased due to the genetic manipulation of PRDX1 (Figure 6).

**Figure 7:**
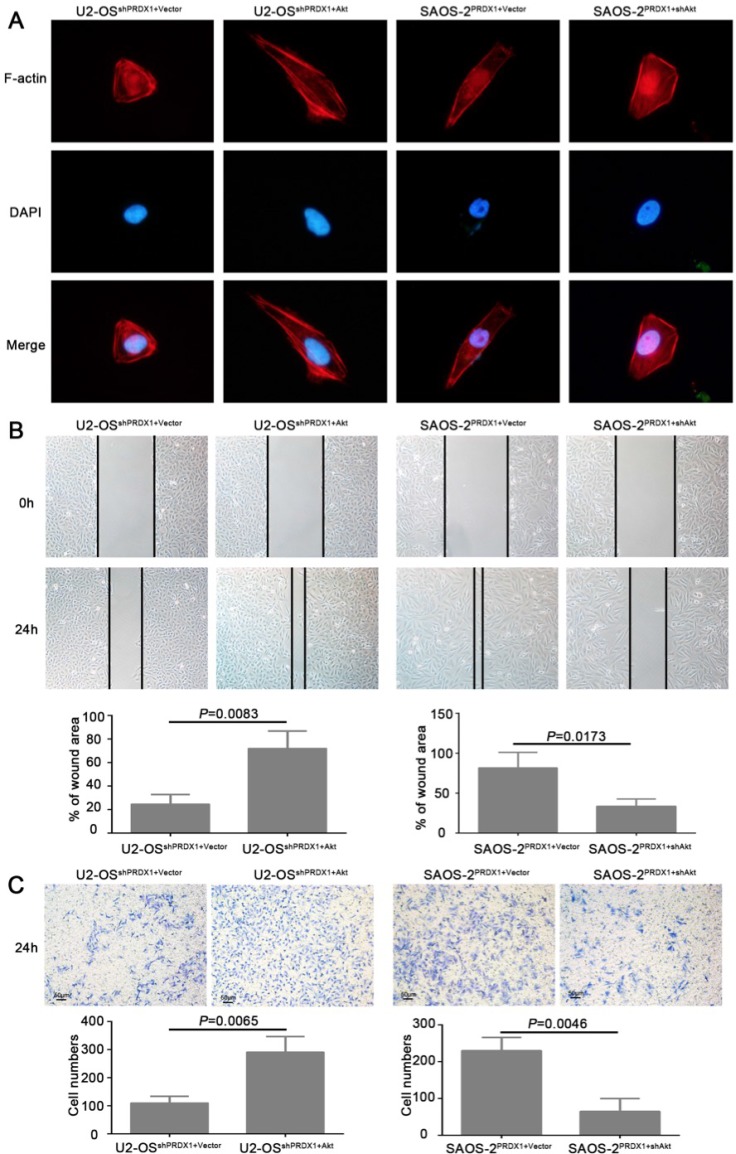
Akt is a critical downstream effector in PRDX1-promoted proliferation in osteosarcoma (**A** and **B**) ectopic expression of Akt increased the proliferation and colony formation of U2-OS^shPRDX1^, while down-regulation of Akt reduced cell proliferation and colony formation of SAOS-2^PRDX1^. (**C**) 3 hours EdU labeling shows that overexpression of Akt in U2-OS^shPRDX1^ restore cell proliferation. Moreover, downregulation of Akt abolished the proliferation enhancement caused by overexpression of PRDX-1 in SAOS-2^PRDX1^ cells.

**Figure 8:**
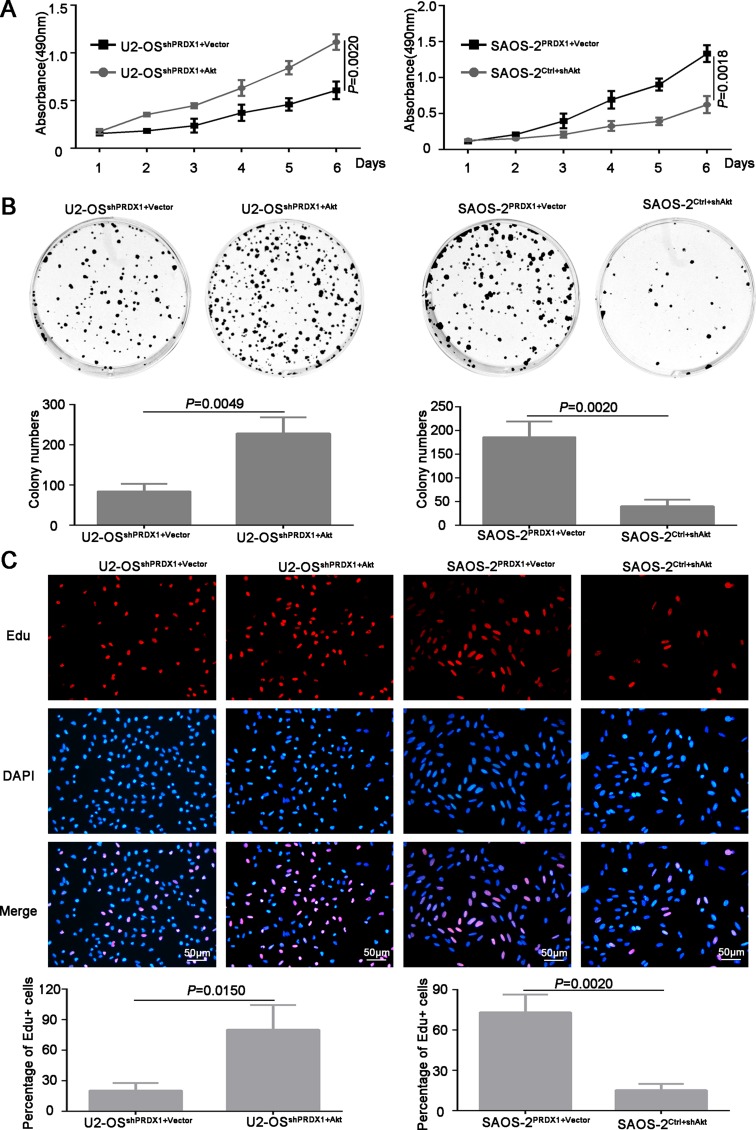
Akt is responsible for PRDX1-related invasion and metastasis (**A**) F-actin Immunofluorescence staining was used to analyze the cell cytoskeleton reorganization. Overexpression or knockdown of Akt could rescue the phenotype induced by genetic manipulating PRDX1. In U2-OS^shPRDX1^ cells, overexpression of Akt resulted the obvious reorganization of cytoskeleton and the cell morphology changed into more fibroblastic-like, while knockdown of Akt in SAOS-2^PRDX1^ induces morphology regressed into cobbles-tone shape. (**B** and **C**) Wound healing and transwell assays showed that the overexpression of Akt in U2-OS^shPRDX1^ could rescue the migration and invasion defects caused by knockdown of PRDX1 (left panel). Moreover, downregulation of Akt abolished the ability of migration and invasion in SAOS-2^PRDX1^ cells (right panel).

To determine whether Akt is the downstream mediator for PRDX1 promoted invasion and metastasis, plasmid containing Akt coding region was transfected into U2-OS^shPRDX1.^ We found that overexpression of Akt could rescue the defects caused by Knockdown of PRDX1 (Figure 7A–7C). Overexpression of Akt in U2-OS^shPrx-1^ increases the migration and invasion (Figure 7B and 7C, left panel). Moreover, downregulation of Akt abolished the effect of Prx-1 overexpression on migration and invasion in SAOS-2^PRDX1^ cells (Figure 7B–7C, right panel). In addition, ectopic expression of Akt increased the proliferation and colony formation of U2-OS^shPRDX1^ (Figure 7A–7C, left panel), while suppression of Akt expression reduced the ability of the proliferation and colony formation of SAOS-2^PRDX1^ (Figure 8A–8C, right panel). Collectively, these results indicate that PRDX1 promotes osteosarcoma invasion and metastasis through enhancing Akt/mTOR pathway.

## DISCUSSION

Although the overall survival (OS) of non-metastatic osteosarcoma has improved significantly with the development of advanced chemotherapy, patients with pulmonary metastasis have poor prognosis [18]. The precise control of metastasis remains unclear. The reactive oxygen species (ROS) play the roles not only in tumor metastasis but also in tumorogenesis [8, 19, 20]. PRDX1 regulate intracellular H_2_O_2_ hemostasis and defense of oxidative at both physiological and pathological situation [21]. Some studies have shown that PRDX1 serve as tumor suppressor in several types of cancers, but other studies prove that PRDX1 may promote tumor growth [22–24]. The biological function of PRDX1 in osteosarcoma is still ambiguous.

In present study, we demonstrated that both PRDX1 mRNA and protein levels elevated significantly in OS tissues and cell lines. PRDX1 level highly correlated with clinicopathological variables, including tumor size, presence of pathological facture, and malignant grade. Moreover, osteosarcoma patients with high PRDX1 level had poor prognosis than those with low level. Notably, expression of PRDX1 significantly increased in lung metastatic tumor, suggesting PRDX1 might play a role in osteosarcoma metastasis.

To validating the function of PRDX1 in osteosarcoma metastasis, we rationally chose Saos-2 for the subsequent gain-of-function and U2-OS for loss-of-function studies base on the endogenous expression level of PRDX1. Our results show that PRDX1 promotes osteosarcoma cell proliferation, invasion *in vitro* and tumor formation *in vivo*, suggesting overexpression of PRDX1 in osteosarcoma related to its development and progression. In addition, overexpression of PRDX1 promotes lung invasion of tumor cells and downregulation of PRDX1 inhibit lung metastasis *in vivo*, which further confirmed our *in vitro* observations that PRDX1 promoted metastasis of osteosarcoma cell. Moreover, we found that PRDX1 increase phosphorylation level of Akt, mTOR and S6K and genetic manipulating of Akt could rescue the defects caused by overexpression or down-regulation of PRDX1.

To conclude, we report that PRDX1 is an important regulator of osteosarcoma carcinogenesis, in which PRDX1 promotes osteosarcoma cell proliferation, migration and metastasis by enhancing phosphorylation of Akt/mTOR. Overexpression of PRDX1 was significantly associated with malignancy and poor prognosis of osteosarcoma. Collectively, our results indicate that PRDX1 function as an oncogene in osteosarcoma and may serve as a promising therapeutic target. This newly identified PRDX1-Akt pathway might argument our knowledge in ROS mediated tumor initiation and promotion.

## MATERIALS AND METHODS

### Patients and tissue specimens

Forty fresh tumor tissues were used to measure the mRNA and protein level of PRDX1. Another two independent cohorts of paraffin-embedded osteosarcoma samples including training cohort (*n* = 115) and validation cohort (*n* = 90) from 2 different centers were used for prognostic study according to REMARK guideline [25]. All FFPE samples were collected from The Second Xiangya Hospital and The Affiliated Cancer Hospital of Xiangya School of Medicine (From June 2007 to April 2010). All procedure was approved by the Ethics Committee of the second Xiangya hospital, Central South University.

All patients were followed-up by experienced clinician. Average follow-up time is 63 months (ranging from 8 to 107 months). Overall survival (OS) was defined as the time between tumor resection and the last follow-up. Patients alive at the end of follow up or dead from causes without sign of recurrence were censored. Disease-free survival (DFS) was calculated from tumor resection to the first evidence of metastasis or/and recurrence.

### Quantitative real-time polymerase chain reaction

Total RNA was isolated from fresh osteosarcoma tissue samples and cell lines by using a TRIzol^®^ Reagent (Invitrogen, Carlsbad, CA) according to the manufacturer protocol. After quantification using a spectrophotometer (Shimadzu, Kyoto, Japan), RNA samples were reversely transcribed into cDNA using a universal cDNA synthesis kit (Toyobo, Osaka, Japan). The cDNA was subjected to quantitative real-time PCR (qRT-PCR) using the SYBR Green PCR Kit (Toyobo) and the assay was performed on an PRISM 7300 Sequence Detection System (Applied Biosystems, CA). Quantitative real-time PCR was done as previous [26]. The PCR parameters were as follows: 40 cycles of 95°C for 5 s and 60°C for 20 s. The primers for PRDX1 were used as follow: Forward, 5′- CCACGGAGATCATTGCTTTCA -3′; Reverse, 5′- AGGTGTATTGACCCATGCTAGAT -3′, the primers for GAPDH: forward, 5′-CCACCCATGGCAAATTCC-3′; reverse, 5′-GATGGGATTTCCATTGATGACA-3′. The relative levels of mRNA were calculated by using the 2^-∆Ct^ method based on the threshold cycle (Ct) values and then normalized to GAPDH. Experiment was repeated at least three times.

### Western blot

Total cellular or tissue protein was extracted by RIPA lysis buffer. The protein concentrations of the lysates were determined according to the bicinchoninic acid (BCA) method using a protein assay kit (Pierce Biotechnology, Rockford, IL). Cell or tissue lysates containing 100 µg proteins were separated by sodium dodecyl sulfate-polyacrylamide gel electrophoresis (SDS-PAGE) and then transferred onto PVDF membranes (Millipore, Billerica, MA). The membranes were blocked with 5% non-fat milk and incubated with primary antibody PRDX1 (Santa Cruz Biotechnology, Santa Cruz, CA), Akt (Cell signaling technology), p-Akt (Cell signaling technology), NF-KB (Abcam, Cambridge, MA), mTOR (Santa Cruz Biotechnology), p-mTOR (Cell signaling technology), S6K (Cell signaling technology), p-S6K (Cell signaling technology) and beta-actin antibody (Sigma, St Louis, MO). The protein complex was detected with enhanced chemiluminescence reagents (Pierce IL, USA).

### Immunohistochemistry and immunofluorescence staining

4 µm sections were de-paraffinized and rehydrated. After antigen retrieval with 1 mM, pH 8.0 EDTA buffer for 10 minutes, endogenous peroxidases were quenched by 0.3% H_2_O_2_ and then blocked with blocking solution. Primary antibody PRDX1 (dilution: 1:200) was developed overnight at 4°C. After washing with PBS (0.3% Triton), the sections were incubated HRP-labeled secondary antibody. Subsequently, the sections were counterstained with hematoxylin solution and mounted with a coverslip. The expression levels of PRDX1 were scored based on signal intensity and percentage of positive cells (16). Briefly, immunostaining score (IS) = SI (staining intensity) × PP (percentage of positive cells). SI was classified as: 0, negative; 1, weak; 2, moderate; 3, strong. PP was defined as 0, 0% positive cells; 1, 0–25% positive cells; 2, 25–50% positive cells; 3, 50–75% positive cells, and 4, 75–100% positive cells. Scoring was performed by two pathologists respectively.

For F-actin visualizing, the cells were incubated with 0.25 mM TRITC-conjugated phalloidin (Sigma) and Nuclei were stained with 4’,6-diamidino-2-phenylindole (DAPI).

### Plasmid construction and generation of stable cell line

hFOB-1.19, MG-63, U2-OS, and SAOS-2, were cultured in RPMI-1640 medium (GIBCO BRL, Gaithersburg, MD) supplemented with 10% fetal bovine serum (FBS; HyClone, Logan, UT) and 1% penicillin/streptomycin in a 5% CO2 atmosphere at 37°C.

PRDX1 short hairpin RNAs (shPRDX1), PRDX1 overexpression plasmid and its scramble control were purchased from GeneChem Company (Shanghai, China). The sequences of the three shRNAs were as follow: PRDX1-shRNA-1, 5’-CCGCTCTGTGGATGAGACTTTGAGACT AG-3’; PRDX1-shRNA-2, 5’- TCAACTGCCAAGTGATT GGTGCTTCTGTG-3’; PRDX1-shRNA-3, 5’- TCTTCGG CAGATCACTGTAAATGACCTCC-3’. SAOS-2 cells were transfected with the shPRDX1 plasmid, and U2-OS cells were transfected with the PRDX1 over-expression plasmid. Cells transfected with scramble vector were used as controls. Cell transfection was done with lipofectamine 3000(Thermo Fisher Scientific). Transfected cells were selected with 3 µg/mL puromycin to generate stable cell line. Down-regulation or overexpression of PRDX1 was confirmed.

### Examination of cell proliferation

Cells were seeded into 96-well plates and incubated with 5% CO_2_ at 37°C. Cell viability was measured by MTT method. Cell proliferation was measured by EdU click method according to the manufacturer’s protocol. All reagents used in this assay was supplied in Cell-Light™ EdU Apollo^®^ 567 Kit (500T)(C10327-1, RiboBio). For colony formation assay, five hundreds cells were seeded into 35 mm dishes and cultured in 5% CO2 for 2 weeks at 37°C. The number of colonies per dish was counted after staining with crystal violet. Only positive colonies (diameter > 40 um) in the dishes were counted. All experiments were repeated at least three times.

### Examination of cell invasion

Cells reach approximates 90% confluence and then cultured in serum-free medium for 24 hours. Three parallel wounds were created using a sterile 10 μl pipette tip and then rinsed with ice-cold PBS. The wound closure was recorded after 24 hours.

1 × 10^5^ cells were seeded into the upper chambers of transwell precoated with matrigel (BD Biosciences, San Jose, CA), while the bottom chambers were filled with 200 µl of regular culture medium. 24 hours later, cells remaining in the upper chamber were removed using a cotton swap. Cells was stained with 0.1% crystal violet, the number of cells in lower membrane of the inserts was counted. All experiments were repeated at least three times.

### Subcutaneous xenograft and tail vein injection

The animal study was approved by the Institutional Animal Care and Use Committee (IACUC) of The Second Xiangya Hospital, Central South University. In briefly, 4 weeks-old BALB/C nude mice were used to measure the metastatic ability and tumor growth *in vivo*. 5 × 10^6^ cells suspended in 0.1 ml saline were injected Subcutaneously or into tail vein (*n* = 6). 30 days after injection, the mice were sacrificed and dissected and volume of tumor size was measured. Lung was harvested and then fixed in 10% buffered formalin, paraffin embedding and serially sectioned. Slices were stained with hematoxylin and eosin (H&E). The number of lung tumor metastatic nodules was counted under the microscope.

### Statistical analysis

All statistical analyses were performed by SPSS18.0 (SPSS Inc., Chicago, IL). Results were presented as mean ± SD and analyzed by using an independent *t* test when the variance was homogeneous. One-Way ANOVA was used to compare the difference among multiple groups. Categorical data were analyzed using Fisher’s exact test. Overall survival (OS) and disease-free survival (DFS) curves were plotted using the Kaplan–Meier method and compared by the log-rank test. *P* < 0.05 was considered statistically significant.
